# PPARalpha-mediated effects of dietary lipids on intestinal barrier gene expression

**DOI:** 10.1186/1471-2164-9-231

**Published:** 2008-05-19

**Authors:** Heleen M de Vogel-van den Bosch, Meike Bünger, Philip J de Groot, Hanneke Bosch-Vermeulen, Guido JEJ Hooiveld, Michael Müller

**Affiliations:** 1 Nutrition, Metabolism and Genomics group, Division of Human Nutrition, Wageningen University, PO Box 8129, NL-6700EV, Wageningen, the Netherlands; 2 Nutrigenomics Consortium, TI Food and Nutrition, P.O. Box 557, NL-6700AN, Wageningen, the Netherlands

## Abstract

**Background:**

The selective absorption of nutrients and other food constituents in the small intestine is mediated by a group of transport proteins and metabolic enzymes, often collectively called 'intestinal barrier proteins'. An important receptor that mediates the effects of dietary lipids on gene expression is the peroxisome proliferator-activated receptor alpha (PPARα), which is abundantly expressed in enterocytes. In this study we examined the effects of acute nutritional activation of PPARα on expression of genes encoding intestinal barrier proteins. To this end we used triacylglycerols composed of identical fatty acids in combination with gene expression profiling in wild-type and PPARα-null mice. Treatment with the synthetic PPARα agonist WY14643 served as reference.

**Results:**

We identified 74 barrier genes that were PPARα-dependently regulated 6 hours after activation with WY14643. For eicosapentaenoic acid (EPA), docosahexaenoic acid (DHA) and oleic acid (OA) these numbers were 46, 41, and 19, respectively. The overlap between EPA-, DHA-, and WY14643-regulated genes was considerable, whereas OA treatment showed limited overlap. Functional implications inferred form our data suggested that nutrient-activated PPARα regulated transporters and phase I/II metabolic enzymes were involved in a) fatty acid oxidation, b) cholesterol, glucose, and amino acid transport and metabolism, c) intestinal motility, and d) oxidative stress defense.

**Conclusion:**

We identified intestinal barrier genes that were PPARα-dependently regulated after acute activation by fatty acids. This knowledge provides a better understanding of the impact dietary fat has on the barrier function of the gut, identifies PPARα as an important factor controlling this key function, and underscores the importance of PPARα for nutrient-mediated gene regulation in intestine.

## Background

The small intestine is the primary site for digestion and selective absorption of nutrients and other food constituents. Absorption of these molecules across the intestinal epithelium is mediated mainly by multiple transmembrane transporters that principally belong to two superfamilies, namely the solute carrier (SLC) and the ATP Binding Cassette (ABC) superfamily of transporters. SLC transporters located at the apical membrane of enterocytes are responsible for the selective uptake of macronutrients, such as di- and tripeptides, hexoses and fatty acids [1]. In contrast, ABC transporters are efflux transporters responsible for the active removal of substances, including nutrients such as cholesterol, regulating their intracellular concentrations [2]. Besides their presence in plasma membranes, SLC and ABC transporters are also located in intracellular organelles, such as mitochondria or peroxisomes, thus regulating intracellular and transcellular solute transport.

In addition, it has become clear that the small intestine is an important metabolic active organ, to a great extend responsible for the first-pass metabolism of nutrients and xenobiotics [3,4]. Numerous metabolic reactions occur in enterocytes, including those typically referred to as phase I and phase II metabolism. Phase I metabolism includes oxidative, peroxidative, and reductive metabolism of endogenous compounds and drugs, mediated by cytochrome P450 isoenzymes (CypP450s) [5]. Phase II metabolism often succeeds phase I metabolism and yields mainly more hydrophilic metabolites, mostly by conjugation, thereby increasing the water solubility of lipophilic compounds. The most important phase II enzymes are sulfotransferases (Sults) [6,7], UDP-glucuronosyltransferases (Ugts) [8], glutathione S-transferases (Gsts) [9,10], N-acetyltransferases (Nats) [11], and epoxide hydrolases (Ephs) [12]. Several ABC transporters are responsible for the excretion of metabolites resulting from phase I and phase II enzymatic transformations [2].

There is increasing interest in the molecular mechanisms underlying the beneficial or adverse effects of foods and food components. Nutrients impact gene expression mainly by activating or suppressing specific transcription factors [13,14]. The most important group of transcription factors involved in mediating the effect of nutrients and their metabolites on gene transcription is the superfamily of nuclear receptors, which consists of 48 members in the human genome [15]. This superfamily is subdivided into six families [16], of which the NR1 family is most relevant to nutrition. One important group of receptors that mediates the effects of dietary fatty acids and its derivatives on gene expression are the Peroxisome Proliferator Activated Receptors (PPARs, NR1C) [16-18]. Three PPAR isotypes, α (NR1C1), δ (also called β) (NR1C2), and γ (NR1C3) are distinguished and characterized by different biological roles. Transcriptional regulation by PPARs requires heterodimerization with the retinoid X receptor (RXR; NR2B) [16,19,20]. When activated by an agonist, the PPAR/RXR heterodimer stimulates transcription via binding to DNA response elements (PPREs) present in and around the promoter of target genes. Besides upregulating gene expression, PPARs are also able to repress transcription by directly interacting with other transcription factors and interfere with their signaling pathways, a mechanism commonly referred to as transrepression [21].

PPARα has been shown to be expressed at a high level in the small intestine [22]. Moreover, the average Western diet contains a high amount of triacylglycerols [23] that are hydrolyzed to monoacylglycerol and free fatty acids before entering the enterocyte [24]. As a result the small intestine is frequently exposed to high levels of natural PPARα agonists. However, currently little is known about the effects of PPARα activation by dietary fats on gene expression in small intestine. Although in several studies small intestinal gene expression was studied after high-fat feeding [25-27], the specific role of PPARα remains to be elucidated.

Here, we take advantage of a unique experimental design using triglycerides composed of identical fatty acids in combination with gene expression profiling to examine the effects of individual dietary fatty acids on intestinal gene expression in mice. By conducting these experiments in wild-type and PPARα -/- mice, and by limiting the exposure time to 6 hours, we were able to elucidate the specific, direct contribution of PPARα in regulating the expression of transport and phase I/II metabolism genes in small intestine.

## Results and discussion

### Effect of acute PPARα activation with OA, EPA, DHA and WY14643

In this study we investigated the role of PPARα on the expression of genes encoding for transport proteins and phase I/II metabolic enzymes, collectively called barrier proteins, which are responsible for the selective absorption and metabolism of food components. Since we are specifically interested in the role PPARα plays in nutrient-mediated gene regulation, we used and compared in our studies 3 natural agonists normally found in the diet [oleic acid (OA, C18:1), eicosapentaenoic acid (EPA, C20:5) and docosahexaenoic acid (DHA, C22:6)]. As reference we used the synthetic agonist WY14643. We analyzed gene expression 6 hours after oral gavage, thus we primarily studied the direct effects of PPARα activation. This time point was chosen because in a pilot experiment we found that after an oral fat load plasma triacylglycerol levels peaked at 2–3 hours post loading and almost returned to basal levels after 6 hours (data not shown), indicating that at that point most of the fat bolus has passed through the enterocytes, and sufficient time remained for transcriptional events to occur. Expression of transport and phase I/II metabolism genes (barrier genes) was studied using microarrays. The Affymetrix GeneChip Mouse Genome 430 2.0 array comprised of 45,038 probesets, representing 16,579 unique genes. Annotation information from Affymetrix was queried to compile a list of transport and phase I/II metabolism genes present on the array (for details, see methods section). This set consisted of 944 probesets, encoding for 529 unique genes, and was used throughout all analyses. We identified 9,426 significantly expressed genes in small intestine, of which 264 were barrier genes.

As expected, of all agonists used in this study, acute treatment with WY14643 provoked the most pronounced response, both with respect to the number of regulated genes and the magnitude of the fold changes. Treatment with WY14643 resulted in the PPARα-specific differential expression of 74 transport and phase I/II metabolism genes (Table 1), of which 32 were expressed at higher levels and 42 genes were reduced in wild-type mice compared to PPARα-null mice (for the full list of regulated genes please see additional data, Table 1). On the other hand, treatment of wild-type and PPARα-null mice with OA identified only 19 PPARα-dependently regulated barrier genes (Table 1). Of these, 13 were induced and 6 repressed (additional data, Table 2). Treatment with EPA and DHA resulted in 46 and 41 PPARα-dependently regulated barrier genes, respectively (Table 1). Activation of PPARα by EPA increased the expression of 32 genes and suppressed 14 genes (additional data, Table 3), whereas for DHA these numbers were 22 and 19, respectively (additional data, Table 4).

**Table 1:** Number of PPARα-dependently regulated genes after treatment with different agonists

	**OA**		**EPA**		**DHA**		**WY14643**	
	
	**All genes**	**Barrier genes**	**All genes**	**Barrier genes**	**All genes**	**Barrier genes**	**All genes**	**Barrier genes**
Number of PPARα-dependent regulated genes	508	19	874	46	894	41	1218	74
Percentage	5.4	7.2	9.3	17.4	9.5	15.5	12.9	28.0

Number of PPARα-dependently regulated genes and corresponding percentages for all genes and the barrier gene set after activation with OA, EPA, DHA, and WY14643. The percentages relate to the total number of expressed genes (9,426) and all barrier genes (264), respectively.

In Bünger et al [22] we reported that under basal (control) conditions only 21 genes were differentially expressed in small intestine of wild-type mice compared to PPARα-null mice. The currently investigated barrier gene set includes 2 of these genes, Slc25a20 and Cyp4a10, which were both expressed at lower levels in the null mice. We found that expression of Slc25a20 was only slightly elevated after acute treatment with WY14643, EPA, and DHA, which indicates that the regulation of Slc25a20 by PPARα may be of less relevance during (nutritional) activation of PPARα. In contrast, the fold induction of Cyp4a10 observed after acute treatment with WY14643, EPA and DHA was much larger than the basal difference, which implies that for this gene activation of PPARα is of importance.

When comparing the list of barrier genes that were PPARα-dependently regulated after acute treatment with WY14643 with that of a long-term (5 day) exposure experiment [22], we found an overlap of 74% (additional data, Table 5). This indicates that short-term regulation evoked with synthetic agonists is maintained for at least 5 days.

Hirai et al [28] very recently reported their study in which they identified seven nutrient and drug transporters that were PPARα-dependently regulated in small intestine after 3 days exposure to two synthetic agonists. In concordance with their data, we found that all except 2 of these transporters were regulated after acute treatment with WY14643 as well. In addition, we observed that these carriers were also PPARα-dependently regulated by DHA and EPA. Like Hirai et al, we did not observe a PPARα-dependent regulation of Pept1 (Slc15a1), the first intestinal nutrient transporter shown to be PPARα-dependently regulated during fasting [28,29].

To characterize the importance of PPARα in controlling expression of small intestinal transporters and phase I/II metabolic enzymes, we compared the fraction of PPARα-dependently regulated genes of the barrier gene set with that observed for all genes (Table 1). For treatment with OA the percentages were 7.2% and 5.4%, respectively, for the set of barrier genes and all expressed genes. This difference was not statistically significant (p = 0.11). However, the other two natural agonists showed a significantly higher percentage of regulated genes for the barrier gene set than for all genes; for EPA this percentage was 9.3% for all genes, whereas 17.4% of the barrier genes were regulated (p < 0.001). For DHA these percentages were 9.5% and 15.5% respectively (p < 0.001). For WY14643 we observed an even larger difference between all genes and the barrier gene set; 12.9% respectively 28.0% of the genes were regulated in a PPARα-dependent manner (p < 0.001). These results imply that PPARα plays an important role in regulating small intestinal gene expression of transporter and phase I/II metabolic enzymes.

### Overlap between OA, EPA, DHA and WY14643 treatment

**Figure 1:**
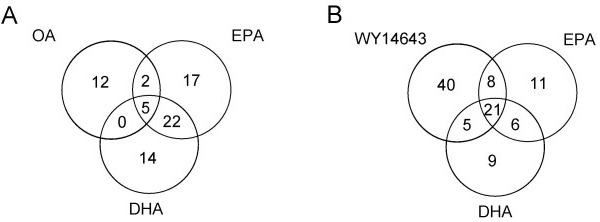
**Overlap of PPARα-dependently regulated genes between the four agonists**. The numbers in the Venn plots represent the numbers of PPARα-dependently regulated genes for each treatment. A) Overlap between OA, EPA and DHA, B) Overlap between EPA, DHA, and WY14643.

We also determined the overlap of PPARα-dependently regulated genes between the different treatments. Most of the genes regulated upon treatment with OA were not regulated by DHA and EPA (Figure 1A) or WY14643 (data not shown). Only four genes, i.e. Slc27a4 (Fatp4), Cyp4F16, Cyp2c65, and Abcd3 were regulated upon all 4 treatments. For these genes additional qRT-PCR analyses were performed, which confirmed the array results (Table 2). There was considerable overlap between the genes affected by EPA, DHA or WY14643 treatment (Figure 1B). These overlapping genes behaved the same in all treatments, i.e. they were either increased or suppressed in wild-type compared to PPARα-null mice upon all treatments. In Table 6 of the additional data the complete list of overlapping genes is presented. It is likely that OA treatment affected fewer genes, because the mice may be adapted to this fatty acid since they were fed a diet based on olive oil three weeks before gavage (for details, see methods section). In addition, it is generally accepted that polyunsaturated fatty acids activate PPARα better than monounsaturated fatty acids [30-33], which is in line with our result that OA activated less genes PPARα-dependently than EPA and DHA. Although the overlap between WY14643-, EPA-, and DHA-regulated genes was high, we still observed differential gene activation between these treatments. The exact mechanism(s) underlying these differences are currently unclear, but we speculate this may be partially due to the differential recruitment of coactivators such as Src-1, Med1, Pgc1α, and p300 by the three agonists [34-37]. Alternatively, hitherto unknown additional signaling routes not shared by the three agonists may exist.

**Table 2:** Confirmation of microarray results

	**WY14643**		**EPA**		**DHA**		**OA**	
**Gene symbol**	**FC (MA)**	**FC (qPCR)**	**FC (MA)**	**FC (qPCR)**	**FC (MA)**	**FC (qPCR)**	**FC (MA)**	**FC (qPCR)**

*Fatp4 (Slc27a4)*	**1.7 **(0.24)	**2.2 **(0.28)*	**1.5 **(0.14)	**1.7 **(0.30)*	**1.4 **(0.14)	**1.5 **(0.09)*	**1.4 **(0.16)	**1.6 **(0.41)*
*Abcd3*	**2.8 **(0.38)	**4.9 **(0.75)*	**1.7 **(0.11)	**2.0 **(0.28)*	**1.8 **(0.08)	**2.7 **(0.50)*	**1.4 **(0.10)	**2.0 **(0.22)*
*Cyp2c65*	**2.6 **(0.34)	**3.7 **(0.69)*	**2.3 **(0.24)	**2.8 **(0.51)*	**2.5 **(0.48)	**3.9 **(1.13)*	**1.7 **(0.23)	**2.7 **(0.94)*
*Cyp4F16*	**1.9 **(0.36)	**2.0 **(0.44)*	**1.5 **(0.14)	**1.3 **(0.30)*	**1.4 **(0.17)	**1.5 **(0.25)*	**1.9 **(0.34)	**2.5 **(0.36)*

Microarray results were confirmed with qPCR. FC = Fold change, MA = microarray, qPCR = quantitative PCR. For the qPCR analysis: mRNA levels were standardized to cyclophilin; expression in the PPARα-null mice was arbitrarily set to 1. Significance was determined by a Bayesian t-test (array data) or unpaired student's *t*-test (qPCR data), * = p-value <0.05. Data are means ± standard error (n = 4–5).

### Functional implications of acute PPARα activation in small intestine

A summary of functional outcomes of PPARα activation by the agonists inferred from our data is presented in table 3. Although in this study we only determined mRNA levels, it has been reported that for the majority of genes the mRNA levels reflect protein abundance very well [38,39]. We therefore allow ourselves to speculate about the functional consequences of nutritional PPARα activation. Nevertheless, these implications should ultimately be evaluated in follow-up studies.

**Table 3:** Overview of regulated processes

**Gene symbol**	**Probeset ID**	**FC WY**	**FC EPA**	**FC DHA**	**FC OA**	**Function**
**Fatty acid oxidation**						
Fatp4 (Slc27a4)	1424441_at	1.7	1.5	1.4	1.4	Apical long chain FA uptake [76]
Cact (Slc25a20)	1423109_s_at	6.8	2.6	2.4	nc	Carnitine cycle in mitochondrial β-oxidation [62]
Octn2 (Slc22a5)	1421848_at	7.7	3.2	4.2	nc	Apical carnitine uptake for mitochondrial β-oxidation [48]
Fatp2 (Slc27a2)	1416316_at	2.2	1.6	nc	nc	Has acyl CoA properties to activate FA for subsequent peroxisomal β-oxidation [86]
Abcd3	1416679_at	2.8	1.7	1.8	1.4	Transport of VLFA over the peroxisomal membrane for peroxisomal β-oxidation [77]
Cyp4a10	1424853_s_at	1447.2	120.2	160.3	nc	ω-oxidation [87]
Cyp4b1	1416194_at	1.7	nc	nc	nc	ω-oxidation [87, 88]
Cyp4f16	1417277_at	1.9	1.5	1.4	1.9	ω-oxidation [89, 90]
G6pt1 (Slc37a4)	1417042_at	-1.5	nc	nc	nc	Glycogenolysis [91]
Nadc1 (Slc13a2)	1418857_at	-2.1	-2.2	nc	nc	Dicarboxylates uptake for Krebs cycle [92]
**Cholesterol flux**						
Npc1l1	1438514_at	-1.5	-1.4	-1.6	nc	Apical cholesterol uptake [93]
Abca1	1450392_at	12.1	3.2	nc	nc	Basolateral cholesterol efflux [56, 58]
**Glucose transport**						
Sglt1 (Slc5a1)	1455431_at	-1.4	nc	nc	nc	Apical glucose uptake [94]
Glut2 (Slc2a2)	1449067_at	-1.4	nc	nc	nc	Basolateral glucose efflux [95]
Glut1 (Slc2a1)	1426599_a_at	-1.5	nc	nc	nc	Glucose transport [95]
Sglt4 (Slc5a9)	1439494_at	-1.7	-1.7	-2.0	nc	Glucose + mannose transport [96]
**Amino acid metabolism**						
Eaac1 (Slc1a1)	1448299_at	-1.4	nc	nc	nc	Apical glutamate uptake [97]
Pat1 (Slc36a1)	1428793_at	nc	-1.6	-1.8	nc	Apical neutral amino acids uptake [98]
Lat2 (Slc7a8)	1417929_at	-1.6	-1.6	-2.0	nc	Basolateral neutral amino acids efflux [99]
Tat1 (Slc16a10)	1436368_at	-1.7	-1.4	-1.8	nc	Basolateral aromatic amino acids efflux [100]
y^+^Lat1 (Slc7a7)	1447181_s_at	1.5	nc	nc	nc	Basolateral cationic amino acids efflux [99]
Aralar1 (Slc25a12)	1428440_at	-1.3	nc	nc	nc	Malate-aspartate shuttle: provides cytosolic aspartate [62]
**Intestinal motility**						
Sert (Slc6a4)	1417150_at	-1.4	-1.2	-1.3	nc	Serotonine uptake [66]
Dat1 (Slc6a3)	1417415_at	nc	1.8	nc	2.6	Dopamine uptake [68]
Nas1 (Slc13a1)	1430804_at	-2.1	-2.0	nc	-2.3	Apical sulphate uptake [101]
**Oxidative stress**						
Dic (Slc25a10)	1416954_at	1.6	1.5	nc	nc	Pyruvate metabolism [62]
Kmcp1 (Slc25a30)	1420836_at	5.5	nc	nc	nc	?
Mct13 (Slc16a13)	1453056_at	10.7	1.5	1.8	nc	?
Svct1 (Slc23a1)	1421912_at	nc	1.7	nc	nc	Apical vitamin C uptake [102]
Svct2 (Slc23a2)	1445589_at	1.4	nc	nc	nc	Basolateral vitamin C uptake [102]
Cyp2c29	1417651_at	nc	3.2	nc	nc	Phase I metabolism
Cyp2c65	1429994_s_at	2.6	2.3	2.5	1.7	Phase I metabolism
Cyp2d22	1419039_at	1.7	1.5	nc	nc	Phase I metabolism
Akr1b8	1448894_at	13.4	3.2	5.1	nc	?
Akr1c12	1422000_at	-1.5	nc	nc	nc	Aldo-ketoreductase activity (Phase II)
Akr1c13	1418672_at	-1.7	nc	nc	nc	Aldo-ketoreductase activity (Phase II)
Ephx1	1422438_at	1.7	1.6	1.9	nc	Epoxide hydrolase activity (Phase II)
Ephx2	1448499_a_at	1.5	1.4	nc	nc	Epoxide hydrolase activity (Phase II)
Gsta1///Gsta2	1421041_s_at	nc	1.2	1.3	nc	Glutathione transferase activity (phase II)
Gsta3	1423436_at	nc	nc	1.8	nc	Glutathione transferase activity (phase II)
Gsta4	1416368_at	nc	nc	1.5	nc	Glutathione transferase activity (phase II)
Gstk1	1452823_at	1.3	1.3	1.5	nc	Glutathione transferase activity (phase II)
Gstm1	1448330_at	nc	1.7	nc	nc	Glutathione transferase activity (phase II)
Gstm3	1427473_at	nc	2.4	2.6	nc	Glutathione transferase activity (phase II)
Gstm4	1424835_at	2.0	1.8	1.8	nc	Glutathione transferase activity (phase II)
Gstm5	1416842_at	-1.3	nc	nc	nc	Glutathione transferase activity (phase II)
Gstm6	1422072_a_at	nc	1.3	nc	nc	Glutathione transferase activity (phase II)
Gstt2	1417883_at	1.2	nc	nc	nc	Glutathione transferase activity (phase II)
Mgst1	1415897_a_at	1.4	1.3	1.2	nc	Glutathione transferase activity (phase II)
Abcg2	1422906_at	1.9	nc	1.3	nc	Apical heme secretion [74]

Overview of PPARα-dependently regulated processes related to transport and phase I/II metabolism in the small intestine. All processes are described in the results and discussion section. FC = fold change, nc = not changed.

### Role of PPARα in intestinal fatty acid oxidation

It is well established that PPARα serves as a master regulator of fatty acid catabolism, which is also apparent from our data [22,40]. Various transporters and phase I enzymes involved in fatty acid uptake and oxidation were PPARα-dependently regulated (Table 3). Although the extent varied somewhat, all 4 agonists regulated long chain fatty acid uptake, mitochondrial and peroxisomal β-oxidation, ω-oxidation, and the metabolism of energy-yielding substrates (glycogenolysis and Krebs cycle). For most genes this regulation was agonist-independent and is consistent with earlier findings [41-43]. It is known that enhanced fatty acid β-oxidation is correlated with reduced severity of inflammatory bowel disease [44]. Furthermore it has been shown that WY14643 treatment caused a reduction of colon injury in a murine DNBS experimental colitis model [45] and that WY14643 treatment might have an anti-inflammatory effect in the small intestine [22]. It has been reported that the expression of Octn2 (Slc22a5), involved in apical carnitine uptake, is induced by WY14643 and clofibrate [46-48]. Here we showed that also EPA and DHA induced expression of Octn2. Recently it has been reported that two functionally relevant polymorphisms in the Octn2 (Slc22a5) gene are associated with increased risk for inflammatory bowel disease [49,50], and that Octn2 expression is decreased in rats with induced inflammatory bowel disease [51]. Taken together, our data imply that nutritional activation of PPARα might be therapeutically valuable for patients with inflammatory bowel disease.

### PPARα regulates intestinal cholesterol flux

Expression of the apical cholesterol uptake transporter Npc1l1 was PPARα-dependently suppressed after treatment with WY14643, EPA, and DHA (Table 3), as also has been observed after fenofibrate treatment [52] and PPARδ activation [53]. It is known that treatment with WY14643 for 5 days induced expression of Abca1 [54]. Here we show that Abca1 is also acutely regulated after PPARα activation. Abca1, which promotes cholesterol efflux at the basolateral membrane to Apo-AI for HDL formation [55-58] was increased after treatment with WY14643 and EPA. Functionally these results suggest that less cholesterol is absorbed from the lumen and more cholesterol is transferred to Apo-A1, resulting in reduced intracellular cholesterol levels in enterocytes. Enterocytes likely compensate for this by increasing the activity of HMG-CoA reductase, as has been reported before [59-61].

### PPARα regulates intestinal nutrient transport and metabolism

Expression of the apical glucose uptake transporter Sglt1 (Slc5a1) and the basolateral glucose efflux transporter Glut2 (Slc2a2) was PPARα-dependently repressed after WY14643 treatment. Furthermore, WY14643, EPA, and DHA all reduced expression of the apical mannose and glucose uptake transporter Sglt4 (Slc5a9), suggesting that PPARα activation results in reduced glucose transport through the intestinal wall. In addition, several transporters involved in the amino acid metabolism were PPARα-dependently regulated (Table 3). Gene expression of small intestinal apical uptake as well as basolateral efflux amino acid transporters was PPARα-dependently suppressed. Furthermore, activation of PPARα reduced expression of Slc25a12 (Aralar1), which is involved in the malate-aspartate shuttle [62]. These effects are in line with data that showed PPARα-mediated downregulation of genes involved in hepatic amino acid metabolism [40,63]. For liver it is suggested that amino acids are conserved for local synthetic processes, including protein and purine synthesis during for instance proliferation [64]. In the small intestine villus length is increased after WY14643 treatment [22], which implies that also in the small intestine amino acids are conserved for local anabolic processes. Taken together, our results suggest that PPARα activation leads to a diminished (neutral) amino acid flux through the enterocyte.

### PPARα regulates intestinal motility

Expression of the serotonin transporter Slc6a4 (Sert) was decreased after treatment with WY14643, EPA, and DHA (Table 3). Serotonin is a neurotransmitter secreted by enterochromaffin cells and is considered to play a key role in functioning of the gut, initiating peristaltic reflex pathways and facilitating propulsive activity [65].

Inactivation of serotonin is crucial to limit its activity, and this is mediated by Sert [66]. The observed reduced expression will result in a diminished activity of Sert, which in turn may increase intestinal motility [66]. Serotonin is detoxified by sulfation inside the enterocyte [67]. The apical sulfate import seems to be reduced as gene expression of the uptake transporter Slc13a1 (Nas1) was decreased. This might be a response to the decreased uptake of serotonin. We also showed that the dopamine transporter Dat1 was PPARα-dependently upregulated after EPA and OA treatment (Table 4). Dopamine increases contractile force of intestinal motility [68], thus more dopamine likely results in increased intestinal motility. Altogether, we believe it is likely that PPARα is involved in regulating intestinal motility. Our data suggest that in feeding conditions PPARα activation may result in speeding-up intestinal motility.

### PPARα diminishes effects of oxidative stress

Oxidative stress results from an imbalance between formation and degradation of pro-oxidants or decreased cellular antioxidant protection mechanisms and may result in increased cell damage and apoptosis [69]. Many genes included in our barrier gene set, such as CypP450s, Gsts, and several Slc transporters, are involved in oxidative stress and were PPARα-dependently regulated. CypP450s induce oxidative stress by oxidative, peroxidative, and reductive metabolism of endogenous compounds and drugs [5], whereas Gsts are involved in the defense against oxidative stress by catalyzing the conjugation of glutathione to a wide variety of endogenous and exogenous electrophilic compounds [70]. Various CypP450 genes were PPARα-dependently upregulated (Table 4), which is in line with data obtained from liver [40]. However, since not all CypP450 genes are expressed in both organs, the regulated genes were not identical. Many Gsts were upregulated by activation with WY14643, EPA, and DHA (Table 3). In addition, various Slc transporters involved in oxidative stress defense were PPARα-dependently upregulated; Dic (Slc25a10), involved in the pyruvate-malate shuttle, citrate-pyruvate shuttle, and gluconeogenesis from pyruvate, is known to protect against oxidative stress [62]; Svct2 (Slc23a2), a basolaterally-located uptake transporter for ascorbic acid [71]; Mct13 (Slc16a13), proposed to play an important role in communicating information on the redox state between cells [72]; Abcg2 (Bcrp1), a secretion transporter of heme and porphyrins located in the apical membrane [73,74]; and Kmcp1 (Slc25a30), probably involved in protection from oxidative damage in situations of increased mitochondrial metabolism [75]. Taken together, we show that many barrier genes involved in defense against oxidative stress were PPARα-dependently upregulated. These data point towards an important role of PPARα in the defense against oxidative stress. In general oxidative stress results in increased cell damage and apoptosis [69] and our data might explain one of the mechanisms by which WY14643 suppresses many genes involved in apoptosis in the small intestine [22].

### Longitudinal distribution of the transcriptional regulation during PPARα activation is not the same for PPARα-dependently regulated genes

**Figure 2:**
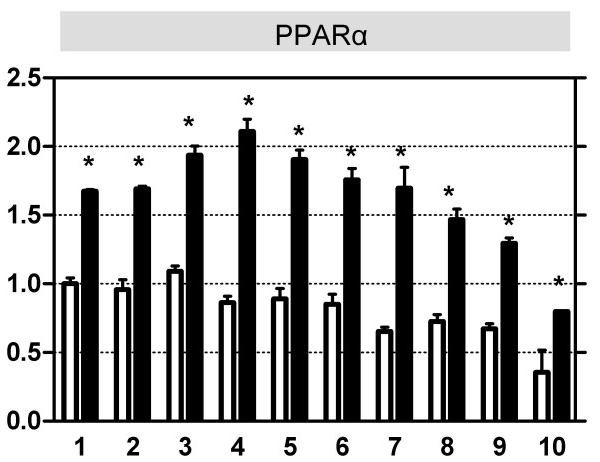
**Expression of PPARα along the longitudinal axis of control and WY14643-treated wild-type mice**. QPCR was used to determine the relative expression levels of PPARα in sections isolated along the proximal-distal axis of the small intestine of wild-type mice that received the control diet (white, open bars), or were acutely treated (6 hr) with WY14643 (black, closed bars) (n = 4 per group). Small intestines were divided into 10 equal parts; part 1 refers to the most proximal part (duodenum), part 10 refers to the most distal (terminal ileum). Messenger RNA levels were standardized to cyclophilin; part 1 of the non-treated mice was arbitrarily set to 1. Significance of control versus treated wild-type mice was determined per segment using an unpaired student's *t*-test. * p-value < 0.05. Data are presented as mean ± standard error.

**Figure 3:**
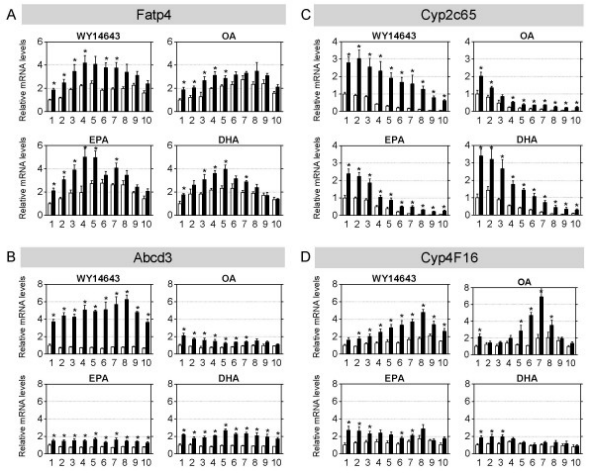
**Expression of PPARα-dependently regulated genes along the longitudinal axis of treated PPARα-null and wild-type mice**. QPCR was used to determine relative expression levels of PPARα-dependently regulated genes in sections isolated along the proximal-distal axis of the small intestine from PPARα-null mice (white, open bars) and wild-type mice (black, closed bars) that were acutely treated (6 hr) with the 4 agonists (n = 4 per group). The small intestine was divided into 10 equal parts; part 1 refers to the most proximal part (duodenum), part 10 refers to the most distal (terminal ileum). Messenger RNA levels were standardized to cyclophilin; part 1 of the PPARα-null mice was arbitrarily set to 1. White bars represent the PPARα-null mice, black bars represent the wild-type mice. Significance of treated WT versus treated KO mice was determined per segment using an unpaired student's *t*-test. * p-value < 0.05. Data are presented as mean ± standard error. A) fatty acid transport protein 4 (Fatp4). B) ATP-binding cassette, sub-family D, member 3 (Abcd3; ALD). C) cytochrome P450, family 2, subfamily c, polypeptide 65 (Cyp2c65). D) cytochrome P450, family 4, subfamily f, polypeptide 16 (Cyp4f16).

Finally we investigated the expression along the proximal-distal axis of PPARα and 4 genes that were PPARα-dependently regulated by all agonists (Figures 2 and 3). Expression was measured with qRT-PCR. For this analysis, the small intestine was divided in 10 equal parts; part 1 represents the proximal side, whereas part 10 represents the most distal end. Expression of PPARα was maximal in duodenum and jejunum, and then gradually declined in ileum, both under basal conditions and after acute activation with WY14643 (Figure 2). For all treatments only the expression pattern in treated wild-type and PPARα-null mice of the 4 genes is reported (Figure 3). Fatp4, Abcd3, Cyp2c65, and Cyp4f16 are all involved in fatty acid metabolism; Fatp4 mediates the apical uptake of long chain fatty acids [76], whereas Abcd3 is involved in the peroxisomal β-oxidation of long chain fatty acids [77]. The human homolog of Cyp2c65 (CYP2C8) metabolizes arachidonic acid and generates epoxygenase products [78]. The rat homolog of Cyp4F16 (Cyp4F5) is involved in ω-oxidation of prostaglandins [79].

In EPA-, DHA- and WY14643-treated mice we observed a similar expression pattern of Fatp4 (Figure 3A), which closely resembled that of PPARα under control and WY14643-activated conditions. In contrast, OA-treated wild-type mice did not show this pattern. In all treatments no significant PPARα-dependent induction of Fatp4 was observed in the distal part of the small intestine. Abcd3 was uniformly induced by all treatments (Figure 3B). Activation with WY14643 revealed a robust, equal induction in all segments in wild-type compared to PPARα-null mice, whereas these were less for the three natural agonists. Cyp2c65 was predominantly expressed in the proximal part of the small intestine, and showed high similarity between agonists (Figure 3C). For each agonist we observed an induction of its expression which was equal along the complete longitudinal axis. Cyp4F16 was uniformly expressed along the proximal-distal axis in treated PPARα-null mice (Figure 3D). However, treatment of wild-type mice with WY14643 and OA shifted the expression of Cyp4F16 to more distal regions, whereas EPA and DHA treatment resulted in significant increased expression in more proximal segments.

Taken together, the data in Figure 3 show that in general all agonists provoke a similar effect on expression of 4 PPARα-dependently regulated genes, and this induction also occurs in more distally-located cells. The latter demonstrates that despite its relatively low expression, PPARα is still able to regulate gene expression.

## Conclusion

In the current study we have identified intestinal barrier genes that were PPARα-dependently regulated after acute activation by fatty acids. The functional outcomes inferred from our data suggest that nutritional-activated PPARα controls processes ranging from fatty acid oxidation and cholesterol-, glucose-, and amino acid-transport and metabolism to intestinal motility and oxidative stress. Altogether, we showed that PPARα has a great impact in controlling the barrier function of the gut, and this underscores the importance of PPARα for nutrient-mediated gene regulation in intestine.

## Methods

### Animals and materials

Pure bred wild-type (129S1/SvImJ) and Pparα-null (129S4/SvJae) mice [80] were bred and housed as described [81]. All animal studies were approved by the Local Committee for Care and Use of Laboratory Animals. The synthetic triacylglycerols trieicosapentaenoin and tridocosahexaenoin were bought from Nu-Chek-Prep, Inc (Elysian, MN), whereas triolein was from Fluka (Zwijndrecht, the Netherlands). These are synthetic triacylglycerols with three identical acyl moieties, namely eicosapentaenoic acid (EPA), docosahexanoic acid (DHA) and oleic acid (OA), which are released as free fatty acids upon digestion in the small intestinal lumen. All three fatty acids have been reported to bind PPARα with varying affinities in the micromolar range [30-33]. WY14643 was obtained from Chemsyn (Lenexa, KS).

### Experimental design and tissue handling

Four months-old male wild-type and PPARα-null mice were used in this study (n = 4–5 per group). Two weeks before the start of the experiment all mice were put on a background diet, which was a modified AIN76A diet (Research diet services, Wijk bij Duurstede, The Netherlands). The AIN76A diet contains 5% w/w corn oil (~10 energy%) [82], which is a relatively low amount of fat. In the current study we replaced the corn oil by the same amount of olive oil (predominantly consisting of oleic acid), since Ren et al [83] demonstrated that an olive oil-rich diet did not regulate established PPARα target genes. The modified AIN76A diet was thus assumed to be a 'poor PPARα-activating' diet, and therefore we hypothesized the number of genes PPARα-dependently regulated by OA would be nominal. However, since the amount of OA in the diet was lower than the amount dosed by gavage (see below), some genes were expected to be regulated.

At the day of the experiment mice were fasted for four hours. At 9 AM mice were dosed by oral gavage with 400 μl of the synthetic triacylglycerols triolein, trieicosapentaenoin, or tridocosahexaenoin, or 400 μl of a 0.1% WY14643 suspension in 0.5% carboxymethyl cellulose (Sigma-Aldrich, Zwijndrecht, the Netherlands). The volume of all doses (400 μl) equalled the maximum recommended volume for gastric gavages for mice [84]. For the fatty acids these doses corresponded to approximately 12.5 g/kg body weight. To put this amount into perspective, data on food intake (not shown) revealed that the mice consumed approximately 4 gram of the modified AIN76A diet per day, which corresponds to approximately 200 mg (6.7 g/kg body weight) of fat. The amount of WY14643 the mice received (approximately 130 mg/kg body weight) was based on previously published short-term study [85].

Six hours after the gavage the mice were anaesthetized with a mixture of isofluorane (1.5%), nitrous oxide (70%) and oxygen (30%). Small intestines were isolated and flushed with ice-cold phosphate-buffer saline and subsequent tissue handlings were performed on ice. Remaining fat and pancreatic tissue was carefully removed from the intestines. For RNA analyses of total tissue, we used full-length small intestine (microarray analyses), or sections obtained after dividing the small intestine into 10 equal parts (studying gene expression distribution along the proximal-distal axis). All small intestinal samples were snap-frozen in liquid nitrogen and stored at -80°C until RNA isolation.

### RNA isolation, Affymetrix GeneChip oligoarray hybridization and scanning, and quantitative real-time PCR

RNA isolation, Affymetrix GeneChip oligoarray hybridization and scanning, and quantitative real-time PCR were performed as described previously [81]. The sequences of primers used in qRT-PCR are available on request. For microarray analyses, RNA was isolated from the full-length small intestine. RNA was hybridized on an Affymetrix GeneChip Mouse Genome 430 2.0 array. This array detects 45,038 transcripts that represent 16,579 known genes. For each experimental group, four or five biological replicates were hybridized for wild-type and PPARα-null mice, and in total 35 arrays were used. Array data have been submitted to the Gene Expression Omnibus, accession number GSE9533.

### Analyses of microarray data

Microarrays were analyzed as described previously [81]. To compile a list of transport and phase I/II metabolism (barrier) genes represented on the array, annotation information from Affymetrix (release of July 2006) was queried for SLC transporters, ABC transporters, CypP450s, the phase II metabolism enzymes glutathione S-transferases, sulfotransferases, epoxide hydrolases, aldo-keto reductases, N-acetyltransferases, and glucuronosyl transferases. Also glutathione reductase, glutathione synthetase, and glutathione peroxidases were included in this set. The final set consisted of 944 probesets, encoding for 529 unique genes. To study significantly expressed genes, only probesets with an expression estimate higher than 32 in either of the 8 experimental groups were selected for further analysis. This cut-off value was based qPCR experiments, because regulation of genes with an expression estimate >32 on the array could all be confirmed by qPCR [81]. The filtering was done after normalization and data analysis. Probesets that had a Bayesian comparison p-value <0.01 were considered to be significantly regulated; no cut-off value of the fold change was used. Of these, probesets that were changed in treated wild-type mice compared to treated PPARα-null mice, were designated PPARα-dependently regulated. QPCR data confirming our array analysis is presented in Table 2 and additional data, Table 7. Differences on the number of regulated genes between gene sets were tested for significance by a one-tailed binominal test. Interpretations of functional outcomes focused on groups of genes that are known to be functionally related (i.e. having a similar function or participating in the same pathway).

## Authors' contributions

MM and GH conceived the study and supervised its design and coordination. The design of the study was set up by HdV and MB. HdV, MB, HB and GH were involved in experimental work. Microarray analysis was performed by HdV, PdG and GH. HdV drafted the manuscript and GH and MM participated in its preparation. All authors have read and approved the final manuscript.

## Supplementary Material

Additional file 1 PPARα-dependently regulated barrier genes upon WY14643 treatment. Click here for file

Additional file 2 PPARα-dependently regulated barrier genes upon OA treatment. Click here for file

Additional file 3 PPARα-dependently regulated barrier genes upon EPA treatment. Click here for file

Additional file 4 PPARα-dependently regulated barrier genes upon DHA treatment. Click here for file

Additional file 5 PPARα-dependently regulated barrier genes after acute (6 hr) and long-term (5 day) treatment with WY14643. Click here for file

Additional file 6 Overlap of PPARα-dependently regulated barrier genes after acute treatment with WY14643, EPA, and DHA. Click here for file

Additional file 7 Additional confirmatory qPCR data. Click here for file
