# Supplementary material for: PPARalpha-mediated effects of dietary lipids on intestinal barrier gene expression
Source: BMC Genomics. 2008 May 19;9:231. doi: 10.1186/1471-2164-9-231 (PMC2408604; doi:10.1186/1471-2164-9-231)
Supplement: Additional file 1 — PPARα-dependently regulated barrier genes upon WY14643 treatment. [file 1471-2164-9-231-S1.pdf]

Additional data, table 1:
PPARα-dependently regulated barrier genes after acute treatment (6hr) with WY14643

Gene symbol	Probe set ID	FC acute	P-value acute	Average WT	Average KO
Slc16a13	1453056_at	10.7	5.9E-07	8.2	4.7
Slc22a5	1440536_at	8.3	5.1E-08	7.9	4.9
Slc22a5	1421848_at	7.7	1.6E-07	10.0	7.0
Slc25a20	1423109_s_at	6.8	1.8E-09	11.2	8.4
Slc22a3	1420444_at	5.8	5.1E-07	5.5	3.0
Slc25a20	1423108_at	5.6	3.4E-08	11.6	9.1
Slc25a30	1420836_at	5.5	8.1E-06	7.1	4.6
Slc22a5	1450395_at	4.7	3.2E-07	11.8	9.6
Slc25a30	1420835_at	4.2	2.4E-05	5.9	3.8
Slc25a30	1450018_s_at	3.4	2.9E-05	5.3	3.5
Slc27a2	1416316_at	2.2	8.2E-06	12.1	11.0
Slc25a4	1455069_x_at	2.1	1.4E-06	10.1	9.0
Slc25a4	1434897_a_at	2.0	1.4E-06	10.4	9.4
Slc25a4	1424562_a_at	1.9	9.9E-07	10.5	9.6
Slc5a6	1435860_at	1.8	1.7E-04	6.2	5.3
Slc27a4	1424441_at	1.7	1.5E-04	10.6	9.8
Slc25a10	1416954_at	1.6	7.0E-05	9.4	8.7
Slc16a1	1415802_at	1.5	3.7E-03	10.0	9.4
Slc7a7	1447181_s_at	1.5	3.4E-03	9.6	9.0
Slc35f5	1452059_at	1.4	7.6E-04	9.8	9.3
Slc23a2	1445589_at	1.4	4.9E-04	6.6	6.2
Slc7a7	1417392_a_at	1.3	4.0E-04	11.1	10.7
Slc25a11	1426586_at	1.2	8.4E-03	10.0	9.7
Slc4a4	1434096_at	-1.3	4.8E-03	9.0	9.3
Slc5a1	1419057_at	-1.3	3.0E-03	12.9	13.3
Slc44a4	1416596_at	-1.3	4.7E-03	9.8	10.2
Slc25a12	1428440_at	-1.3	2.1E-03	7.6	8.0
Slc19a1	1420138_at	-1.3	2.3E-03	8.0	8.4
Slc35d2	1453300_at	-1.3	4.0E-03	6.8	7.2
Slc46a1	1426715_s_at	-1.3	5.9E-04	10.8	11.3
Slc46a1	1426714_at	-1.3	9.8E-04	10.8	11.2
Slco2b1	1433933_s_at	-1.3	6.3E-04	7.9	8.3
Slc30a1	1436164_at	-1.3	8.9E-03	7.8	8.3
Slc17a5	1429116_at	-1.4	2.7E-03	6.9	7.4
Slc19a2	1441315_s_at	-1.4	5.5E-04	7.5	8.0
Slc39a14	1427035_at	-1.4	4.6E-03	9.5	9.9
Slc12a6	1449878_a_at	-1.4	4.1E-03	8.5	8.9
Slc6a4	1417150_at	-1.4	2.0E-03	9.6	10.1
Slc5a1	1455431_at	-1.4	5.1E-04	13.0	13.5
Slc2a2	1449067_at	-1.4	2.2E-03	11.4	11.9
Slc1a1	1448299_at	-1.4	4.0E-03	7.6	8.1
Slc6a7	1455469_at	-1.4	3.7E-03	5.3	5.9
Slc13a1	1431379_a_at	-1.5	2.8E-03	11.1	11.6
Slc39a8	1416832_at	-1.5	8.5E-04	6.3	6.8
Slc2a1	1426599_a_at	-1.5	3.1E-03	6.3	6.9
Slc5a9	1426634_at	-1.5	1.8E-04	6.9	7.5

Slc30a2	1427339_at	-1.5	3.6E-03	7.6	8.2
Slc37a4	1417042_at	-1.5	5.8E-05	9.5	10.1
Slc7a8	1417929_at	-1.6	2.8E-05	8.0	8.6
Slc5a4a	1421637_at	-1.7	6.8E-03	8.0	8.7
Slc16a10	1436368_at	-1.7	3.6E-06	9.1	9.9
Slc5a9	1439494_at	-1.7	8.1E-05	7.0	7.7
Slc20a1	1448568_a_at	-1.7	1.4E-03	8.6	9.4
Slc16a9	1429727_at	-1.7	6.7E-05	9.1	9.8
Slc16a9	1429726_at	-1.9	3.6E-04	6.9	7.8
Slc13a1	1430804_at	-2.1	5.4E-03	7.8	8.9
Slc13a2	1418857_at	-2.1	1.2E-05	10.8	11.8
Slc4a7	1457528_at	-2.3	3.8E-04	9.0	10.2
Slc37a2	1452492_a_at	-3.3	1.4E-04	3.9	5.6
Npc1L1	1438514_at	-1.5	4.7E-04	11.7	12.3
Cyp4a10	1424853_s_at	1447.2	8.1E-14	12.3	1.8
Cyp2c65	1429994_s_at	2.6	6.4E-07	12.5	11.2
Cyp4f16	1430172_a_at	1.9	8.5E-05	10.7	9.7
Cyp4f16	1417277_at	1.9	4.4E-05	10.3	9.3
Cyp2d22	1419039_at	1.7	3.3E-04	7.9	7.1
Cyp4b1	1416194_at	1.7	6.1E-04	11.7	10.9
Cyp2d22	1419040_at	1.5	4.4E-03	7.6	7.0
Cyp3a13	1419523_at	-1.2	5.2E-03	12.7	13.0
Cyp2j6	1417952_at	-1.4	1.2E-03	10.4	10.8
Cyp2s1	1428283_at	-1.4	4.6E-04	8.7	9.2
Akr1b8	1448894_at	13.4	1.6E-06	7.9	4.2
Gstm4	1424835_at	2.0	1.5E-04	5.5	4.5
Ephx1	1422438_at	1.7	1.9E-03	9.6	8.8
Ephx2	1448499_a_at	1.5	3.3E-04	12.5	11.9
Ugt1a2 /// Ugt1a6a /// Ugt1a10 /// Ugt1a7c /// Ugt1a5 /// Ugt1a9 /// Ugt1a6b /// Ugt1a1	1426261_s_at	1.5	6.5E-04	12.5	11.9
Ugt1a2 /// Ugt1a6a /// Ugt1a10 /// Ugt1a7c /// Ugt1a5 /// Ugt1a9 /// Ugt1a6b /// Ugt1a1	1424783_a_at	1.5	9.7E-05	12.2	11.6
Mgst1	1415897_a_at	1.4	2.3E-04	12.6	12.0
Gstk1	1452823_at	1.3	3.6E-03	10.5	10.1
Ugt1a2	1426260_a_at	1.3	4.7E-03	12.9	12.6
Gstt2	1417883_at	1.2	4.2E-03	9.4	9.1
Ugt2b34	1427961_s_at	-1.3	6.1E-03	12.0	12.3
Gstm5	1416842_at	-1.3	2.7E-03	8.7	9.0
Sult1b1	1418940_at	-1.3	1.4E-03	11.8	12.2
Ugt2a3	1450133_at	-1.4	6.4E-04	10.1	10.6

Ugt2b34	1427960_at	-1.5	3.5E-04	11.6	12.2
Akr1c12	1422000_at	-1.5	4.5E-05	10.6	11.2
Akr1c12	1450455_s_at	-1.7	9.4E-06	12.1	12.9
Akr1c13	1418672_at	-1.7	5.6E-05	11.2	11.9
Akr1c19	1455454_at	-1.9	2.1E-04	9.4	10.3
Abca1	1450392_at	12.1	3.1E-05	6.9	3.3
Abca1	1421839_at	10.9	4.2E-07	7.9	4.5
Abca1	1421840_at	5.1	2.5E-06	10.3	8.0
Abcd3	1416679_at	2.8	1.6E-07	13.1	11.6
Abcg2	1422906_at	1.9	4.2E-06	11.8	10.8
Abcd1	1418838_at	1.2	7.2E-03	9.4	9.1

Presented are all PPARα-dependently regulated barrier genes in the small intestine after acute WY14643 treatment. Microarray analysis was performed as described in materials and methods. Listed are the gene symbols, corresponding Affymetrix probeset identifiers, fold changes (FC), the comparison p-values as determined in wild-type mice, and the average log2 transformed expression estimates of the probesets in wild-type (WT) and PPARα-null (KO) mice. A positive FC value indicates a gene expressed at higher levels, whereas a negative FC indicates a gene expressed at lower levels in the treated wild-type mice compared to control. Note that all these genes were not regulated in the PPARα-null mice.
