# Supplementary material for: PPARalpha-mediated effects of dietary lipids on intestinal barrier gene expression
Source: BMC Genomics. 2008 May 19;9:231. doi: 10.1186/1471-2164-9-231 (PMC2408604; doi:10.1186/1471-2164-9-231)
Supplement: Additional file 2 — PPARα-dependently regulated barrier genes upon OA treatment. [file 1471-2164-9-231-S2.pdf]

Additional data, table 2:

PPARα-dependently regulated barrier genes after acute treatment (6hr) with oleic acid (OA)

Gene symbol	Probe set ID	FC	P-value	Average WT	Average KO
Slc6a3	1417415_at	2.6	3.0E-03	7.4	6.1
Slc22a3	1420444_at	1.9	6.2E-04	4.4	3.4
Slc20a2	1457302_at	1.6	2.1E-03	8.8	8.1
Slc5a4b	1422757_at	1.5	6.5E-03	8.7	8.2
Slc27a4	1424441_at	1.4	4.1E-03	10.4	10.0
Slc30a10	1438751_at	1.4	4.7E-03	8.3	7.8
Slc39a6	1424674_at	1.3	5.5E-03	6.6	6.2
Slc35c2	1420054_s_at	1.3	5.1E-03	9.2	8.9
Slc7a6os	1429596_at	1.3	6.3E-03	5.5	5.1
Slc40a1	1448566_at	-1.4	5.1E-03	8.8	9.3
Slc13a1	1431379_a_at	-1.5	7.6E-04	11.1	11.7
Slc40a1	1417061_at	-1.5	3.0E-03	9.2	9.8
Slc37a2	1452492_a_at	-1.8	1.9E-03	3.6	4.5
Slc16a9	1454104_a_at	-1.9	7.1E-03	5.8	6.7
Slc16a9	1429726_at	-2.3	4.1E-03	5.8	7.0
Slc13a1	1430804_at	-2.3	6.9E-04	7.9	9.1
Cyp4f16	1417277_at	1.9	1.9E-05	10.7	9.7
Cyp4f16	1430172_a_at	1.9	4.2E-04	11.0	10.1
Cyp2c65	1429994_s_at	1.7	9.6E-03	11.6	10.9
Gss	1448273_at	1.3	4.1E-03	9.3	8.9
Gal3st1	1454078_a_at	-1.4	9.6E-03	7.6	8.0
Abcd3	1416679_at	1.4	1.5E-03	12.5	12.0
Tap1	1416016_at	-1.4	1.0E-03	6.9	7.5

Presented are all PPARα-dependently regulated barrier genes in the small intestine after acute OA (oleic acid) treatment. Microarray analysis was performed as described in materials and methods. Listed are the gene symbols, corresponding Affymetrix probeset identifiers, fold changes (FC), the comparison p-values as determined in wild-type mice, and the average log2 transformed expression estimates of the probesets in wild-type (WT) and PPARα-null (KO) mice. A positive FC value indicates a gene expressed at higher levels, whereas a negative FC indicates a gene expressed at lower levels in the treated wild-type mice compared to control. Note that all these genes were not regulated in the PPARα-null mice.
