# Supplementary material for: PPARalpha-mediated effects of dietary lipids on intestinal barrier gene expression
Source: BMC Genomics. 2008 May 19;9:231. doi: 10.1186/1471-2164-9-231 (PMC2408604; doi:10.1186/1471-2164-9-231)
Supplement: Additional file 3 — PPARα-dependently regulated barrier genes upon EPA treatment. [file 1471-2164-9-231-S3.pdf]

Additional data, table 3:
PPARα-dependently regulated barier genes after acute treatment (6hr) with eicosapentaenoic acid

Gene symbol	Probe set ID	FC	P-value	Average WT	Average KO
Slc22a5	1421848_at	3.2	1.5E-06	8.2	6.5
Slc25a20	1423109_s_at	2.6	1.2E-08	10.1	8.7
Slc19a3	1450348_at	2.4	1.3E-04	5.4	4.2
Slc25a20	1423108_at	2.3	1.6E-08	10.6	9.4
Slc22a5	1440536_at	2.3	2.4E-04	4.9	3.7
Slc22a5	1450395_at	2.0	4.6E-07	10.2	9.2
Slc6a3	1417415_at	1.8	4.7E-03	4.9	4.1
Slc23a1	1421912_at	1.7	1.9E-03	7.8	7.1
Slc27a2	1416316_at	1.6	4.8E-04	10.6	9.9
Slc25a10	1416954_at	1.5	2.1E-04	9.2	8.6
Slc16a13	1453056_at	1.5	8.7E-03	5.1	4.5
Slc27a4	1424441_at	1.5	7.2E-05	10.3	9.7
Slc25a10	1416955_at	1.4	9.2E-03	7.7	7.2
Slc20a2	1457302_at	1.4	7.6E-04	8.0	7.4
Slc35a3	1429649_at	1.3	5.9E-03	9.1	8.6
Slc23a2	1442983_at	1.3	5.7E-03	7.0	6.7
Slc20a2	1434235_at	1.3	6.0E-03	7.3	7.0
Slc25a4	1455069_x_at	1.2	1.5E-03	9.3	9.0
Slc25a4	1424562_a_at	1.2	3.1E-03	9.8	9.6
Slc6a4	1417150_at	-1.2	6.1E-03	9.7	10.0
Slc12a7	1418257_at	-1.2	9.2E-03	9.1	9.3
Slc15a1	1419343_at	-1.3	4.2E-03	8.6	9.0
Slc30a2	1427339_at	-1.4	1.6E-03	7.5	8.0
Slc16a10	1436368_at	-1.4	6.5E-03	8.7	9.2
Slc5a9	1426634_at	-1.4	2.3E-03	6.3	6.9
Slc4a4	1426432_a_at	-1.5	7.1E-03	4.9	5.5
Slc7a8	1417929_at	-1.6	8.5E-04	7.0	7.7
Slc36a1	1428793_at	-1.6	4.6E-03	8.3	9.0
Slc5a9	1439494_at	-1.7	8.2E-04	6.4	7.2
Slc11a2	1441709_at	-1.7	1.4E-04	5.4	6.2
Slc13a1	1430804_at	-2.0	3.0E-04	9.4	10.4
Slc34a2	1416854_at	-2.7	4.6E-03	7.4	8.9
Npc1L1	1438514_at	-1.4	3.6E-04	11.4	11.9
Cyp4a10	1424853_s_at	120.2	4.7E-12	8.8	1.9
Cyp2c29	1417651_at	3.2	4.8E-04	6.4	4.7
Cyp2c65	1429994_s_at	2.3	2.2E-08	12.2	11.0
Cyp4f16	1417277_at	1.5	1.9E-04	9.8	9.2
Cyp4f16	1430172_a_at	1.5	2.4E-03	10.2	9.6
Cyp2d22	1419039_at	1.5	2.0E-04	6.7	6.2
Cyp51	1450646_at	-1.4	6.8E-04	9.2	9.6
Akr1b8	1448894_at	3.2	1.9E-06	7.6	5.9
Gstm3	1427473_at	2.4	7.5E-05	7.3	6.1
Hs3st1	1423450_a_at	2.1	5.9E-03	6.7	5.6
Gstm3	1427474_s_at	2.0	7.8E-05	10.3	9.3

Gstm4	1424835_at	1.8	1.8E-05	5.0	4.2
Gstm1	1448330_at	1.7	3.9E-04	9.0	8.3
Ephx1	1422438_at	1.6	2.1E-05	8.9	8.2
Gstm1	1416416_x_at	1.6	3.9E-04	9.1	8.4
Ephx2	1448499_a_at	1.4	3.4E-03	11.6	11.1
Gstm6	1422072_a_at	1.3	4.5E-03	8.1	7.7
Ugt1a2 /// Ugt1a6a /// Ugt1a10 /// Ugt1a7c /// Ugt1a5 /// Ugt1a9 /// Ugt1a6b /// Ugt1a1	1426261_s_at	1.3	1.9E-03	11.9	11.6
Mgst1	1415897_a_at	1.3	9.2E-04	12.3	11.9
Gstk1	1452823_at	1.3	4.3E-04	10.4	10.0
Ugt1a2 /// Ugt1a6a /// Ugt1a10 /// Ugt1a7c /// Ugt1a5 /// Ugt1a9 /// Ugt1a6b /// Ugt1a1	1424783_a_at	1.2	7.6E-03	11.6	11.3
Gsta1 /// Gsta2	1421041_s_at	1.2	6.0E-03	13.8	13.6
Abca1	1450392_at	3.2	1.1E-04	5.1	3.4
Abca1	1421839_at	2.9	1.9E-04	6.1	4.6
Abca1	1421840_at	2.1	3.6E-06	9.0	7.9
Abcd3	1416679_at	1.7	3.4E-07	12.4	11.7

Presented are all PPARα-dependently regulated barrier genes in the small intestine after acute EPA (eicosapentaenoic acid) treatment. Microarray analysis was performed as described in materials and methods. Listed are the gene symbols, corresponding Affymetrix probeset identifiers, fold changes (FC), the comparison p-values as determined in wild-type mice, and the average log2 transformed expression estimates of the probesets in wild-type (WT) and PPARα-null (KO) mice. A positive FC value indicates a gene expressed at higher levels, whereas a negative FC indicates a gene expressed at lower levels in the treated wild-type mice compared to control. Note that all these genes were not regulated in the PPARα-null mice.
