# Supplementary material for: PPARalpha-mediated effects of dietary lipids on intestinal barrier gene expression
Source: BMC Genomics. 2008 May 19;9:231. doi: 10.1186/1471-2164-9-231 (PMC2408604; doi:10.1186/1471-2164-9-231)
Supplement: Additional file 4 — PPARα-dependently regulated barrier genes upon DHA treatment. [file 1471-2164-9-231-S4.pdf]

Additional data table 4:
PPARα-dependently regulated barrier genes after acute treatment (6hr) with docosahexanoic acid

Gene symbol	Probe set ID	FC	P-value	Average WT	Average KO
Slc22a5	1421848_at	4.2	4.0E-07	8.5	6.4
Slc22a5	1440536_at	3.1	6.0E-07	5.7	4.1
Slc22a5	1450395_at	2.7	5.3E-08	10.7	9.2
Slc25a20	1423109_s_at	2.4	1.4E-04	10.2	9.0
Slc25a20	1423108_at	2.4	1.2E-05	10.9	9.6
Slc16a13	1453056_at	1.8	1.0E-04	5.3	4.4
Slc20a2	1457302_at	1.4	8.3E-04	7.8	7.3
Slc27a4	1424441_at	1.4	2.1E-03	10.3	9.9
Slc4a4	1434096_at	-1.3	3.4E-03	8.9	9.3
Slc6a4	1417150_at	-1.3	4.1E-04	9.7	10.1
Slc19a1	1448132_at	-1.4	2.2E-03	7.3	7.8
Slc4a4	1452071_at	-1.4	9.2E-03	8.2	8.7
Slc9a6	1435009_at	-1.4	7.9E-03	4.4	4.9
Slc44a4	1416596_at	-1.5	8.8E-04	9.7	10.3
Slc6a8	1417116_at	-1.5	4.7E-04	10.7	11.3
Slc4a4	1426432_a_at	-1.5	3.8E-03	5.1	5.7
Slc4a4	1421225_a_at	-1.5	7.2E-03	7.0	7.6
Slc30a2	1427339_at	-1.6	3.5E-04	7.5	8.2
Slc17a5	1429116_at	-1.7	1.9E-04	6.1	6.9
Slc16a10	1436368_at	-1.8	6.3E-03	8.6	9.4
Slc36a1	1428793_at	-1.8	3.9E-03	8.3	9.1
Slc5a9	1426634_at	-1.8	2.5E-03	6.1	7.0
Slc7a8	1417929_at	-2.0	9.4E-04	7.3	8.3
Slc5a9	1439494_at	-2.0	1.5E-03	6.3	7.3
Slc13a2	1418857_at	-2.2	9.5E-04	10.3	11.4
Slc5a12	1437755_at	-3.8	4.7E-03	7.2	9.1
Slc34a2	1416854_at	-5.0	2.2E-03	5.2	7.6
Npc1L1	1438514_at	-1.4	5.7E-03	11.3	11.7
Cyp4a10	1424853_s_at	160.3	7.9E-11	9.4	2.1
Cyp2c65	1429994_s_at	2.5	1.9E-06	12.3	11.0
Cyp4f16	1417277_at	1.4	1.8E-03	9.7	9.2
Akr1b8	1448894_at	5.1	2.9E-03	8.6	6.3
Hs3st1	1423450_a_at	2.7	3.5E-04	5.5	4.1
Gsta2	1421040_a_at	2.7	2.0E-06	7.8	6.3
Gstm3	1427473_at	2.6	1.4E-04	8.0	6.6
Gstm3	1427474_s_at	2.1	2.2E-04	10.9	9.8
Ephx1	1422438_at	1.9	1.5E-04	9.0	8.1
Gstm4	1424835_at	1.8	2.7E-03	5.1	4.3
Gsta3	1423436_at	1.8	5.4E-03	6.4	5.5
Gstk1	1452823_at	1.5	3.7E-04	10.3	9.8
Gsta4	1416368_at	1.5	3.3E-03	12.2	11.6
Gsta1 /// Gsta2	1421041_s_at	1.3	1.3E-03	14.0	13.6
Ugt1a2 /// Ugt1a6a /// Ugt1a10 ///	1424783_a_at	1.3	3.6E-03	11.8	11.4

Ugt1a7c ///<br>Ugt1a5 ///<br>Ugt1a9 ///<br>Ugt1a6b ///<br>Ugt1a1					
Ugt1a2 ///<br>Ugt1a6a ///<br>Ugt1a10 ///<br>Ugt1a7c ///<br>Ugt1a5 ///<br>Ugt1a9 ///<br>Ugt1a6b ///<br>Ugt1a1	1426261_s_at	1.3	9.2E-03	12.1	11.7
Mgst1	1415897_a_at	1.2	3.9E-03	12.4	12.1
Ndst2	1417931_at	-1.6	2.6E-03	4.4	5.1
Sult1a1	1427345_a_at	-1.6	8.3E-03	6.3	7.0
Abcd3	1416679_at	1.8	7.5E-07	12.6	11.7
Abce1	1442071_at	1.7	3.5E-03	6.3	5.6
Abcg2	1422906_at	1.3	2.3E-03	11.2	10.8
Abca8a	1427371_at	-1.3	7.4E-03	7.1	7.5

Presented are all PPARα-dependently regulated barrier genes in the small intestine after acute DHA (docosahexanoic acid) treatment. Microarray analysis was performed as described in materials and methods. Listed are the gene symbols, corresponding Affymetrix probeset identifiers, fold changes (FC), the comparison p-values as determined in wild-type mice, and the average log2 transformed expression estimates of the probesets in wild-type (WT) and PPARα-null (KO) mice. A positive FC value indicates a gene expressed at higher levels, whereas a negative FC indicates a gene expressed at lower levels in the treated wild-type mice compared to control. Note that all these genes were not regulated in the PPARα-null mice.

.
