# Supplementary material for: PPARalpha-mediated effects of dietary lipids on intestinal barrier gene expression
Source: BMC Genomics. 2008 May 19;9:231. doi: 10.1186/1471-2164-9-231 (PMC2408604; doi:10.1186/1471-2164-9-231)
Supplement: Additional file 5 — PPARα-dependently regulated barrier genes after acute (6 hr) and long-term (5 day) treatment with WY14643. [file 1471-2164-9-231-S5.pdf]

Additional data, table 5:
PPARα-dependently regulated barrier genes after acute (6hr) and long-term (5 day) treatment with WY14643

Gene symbol	Probe set ID	FC WY acute	P-value acute	FC WY 5 days	P-value 5 days
Slc16a13	1453056_at	10.7	5.90E-07	13.8	7.37E-10
Slc22a5	1440536_at	8.3	5.10E-08	11.7	4.24E-09
Slc22a5	1421848_at	7.7	1.60E-07	7.1	4.29E-10
Slc25a20	1423109_s_at	6.8	1.80E-09	6.6	6.48E-10
Slc22a3	1420444_at	5.8	5.10E-07	30.5	9.46E-09
Slc25a20	1423108_at	5.6	3.40E-08	5.5	3.33E-10
Slc25a30	1420836_at	5.5	8.10E-06	7.0	1.56E-09
Slc22a5	1450395_at	4.7	3.20E-07	5.2	2.10E-10
Slc25a30	1420835_at	4.2	2.40E-05	6.6	1.06E-09
Slc25a30	1450018_s_at	3.4	2.90E-05	5.7	5.81E-07
Slc27a2	1416316_at	2.2	8.20E-06	3.9	5.26E-09
Slc25a4	1455069_x_at	2.1	1.40E-06	3.8	2.99E-08
Slc25a4	1434897_a_at	2	1.40E-06	3.6	1.56E-09
Slc25a4	1424562_a_at	1.9	9.90E-07	3.5	9.78E-08
Slc5a6	1435860_at	1.8	1.70E-04	-1.2	3.69E-01
Slc27a4	1424441_at	1.7	1.50E-04	1.5	8.93E-05
Slc25a10	1416954_at	1.6	7.00E-05	1.6	6.73E-04
Slc16a1	1415802_at	1.5	3.70E-03	1.0	7.21E-01
Slc7a7	1447181_s_at	1.5	3.40E-03	1.3	1.69E-02
Slc35f5	1452059_at	1.4	7.60E-04	1.3	4.50E-03
Slc23a2	1445589_at	1.4	4.90E-04	2.0	7.99E-05
Slc7a7	1417392_a_at	1.3	4.00E-04	1.2	1.98E-02
Slc25a11	1426586_at	1.2	8.40E-03	1.3	7.07E-03
Slc4a4	1434096_at	-1.3	4.80E-03	-1.8	3.27E-06
Slc5a1	1419057_at	-1.3	3.00E-03	-1.4	1.26E-02
Slc44a4	1416596_at	-1.3	4.70E-03	-1.8	1.10E-06
Slc25a12	1428440_at	-1.3	2.10E-03	1.1	3.84E-01
Slc19a1	1420138_at	-1.3	2.30E-03	1.1	5.25E-01
Slc35d2	1453300_at	-1.3	4.00E-03	1.1	1.46E-01
Slc46a1	1426715_s_at	-1.3	5.90E-04	-1.3	8.81E-03
Slc46a1	1426714_at	-1.3	9.80E-04	-1.2	2.24E-02
Slco2b1	1433933_s_at	-1.3	6.30E-04	1.1	1.63E-01
Slc30a1	1436164_at	-1.3	8.90E-03	-1.3	1.57E-02
Slc17a5	1429116_at	-1.4	2.70E-03	-1.5	5.28E-04
Slc19a2	1441315_s_at	-1.4	5.50E-04	1.3	8.70E-03
Slc39a14	1427035_at	-1.4	4.60E-03	-1.3	3.91E-03
Slc12a6	1449878_a_at	-1.4	4.10E-03	-1.2	1.47E-01
Slc6a4	1417150_at	-1.4	2.00E-03	-1.5	7.81E-04
Slc5a1	1455431_at	-1.4	5.10E-04	-1.4	1.51E-03
Slc2a2	1449067_at	-1.4	2.20E-03	-1.4	5.11E-04
Slc1a1	1448299_at	-1.4	4.00E-03	-1.7	2.45E-06
Slc6a7	1455469_at	-1.4	3.70E-03	-1.0	6.50E-01
Slc13a1	1431379_a_at	-1.5	2.80E-03	-1.5	1.77E-02
Slc39a8	1416832_at	-1.5	8.50E-04	-0.9	5.08E-01

Slc2a1	1426599_a_at	-1.5	3.10E-03	-2.3	7.24E-07
Slc5a9	1426634_at	-1.5	1.80E-04	-1.8	1.25E-03
Slc30a2	1427339_at	-1.5	3.60E-03	-1.7	4.17E-05
Slc37a4	1417042_at	-1.5	5.80E-05	-1.0	8.74E-01
Slc7a8	1417929_at	-1.6	2.80E-05	-2.0	8.84E-05
Slc5a4a	1421637_at	-1.7	6.80E-03	-3.8	2.55E-07
Slc16a10	1436368_at	-1.7	3.60E-06	-1.6	1.01E-03
Slc5a9	1439494_at	-1.7	8.10E-05	-1.6	2.22E-04
Slc20a1	1448568_a_at	-1.7	1.40E-03	-2.1	6.60E-05
Slc16a9	1429727_at	-1.7	6.70E-05	-0.5	3.40E-03
Slc16a9	1429726_at	-1.9	3.60E-04	-0.6	1.60E-02
Slc13a1	1430804_at	-2.1	5.40E-03	-2.1	1.28E-04
Slc13a2	1418857_at	-2.1	1.20E-05	-1.1	4.22E-01
Slc4a7	1457528_at	-2.3	3.80E-04	-2.3	4.96E-05
Slc37a2	1452492_a_at	-3.3	1.40E-04	-1.3	1.23E-02
Npc1l1	1438514_at	-1.5	4.70E-04	-2.3	2.43E-07
Cyp4a10	1424853_s_at	1447.2	8.10E-14	1170.0	1.97E-17
Cyp2c65	1429994_s_at	2.6	6.40E-07	2.3	1.07E-07
Cyp4f16	1430172_a_at	1.9	8.50E-05	-1.3	8.25E-03
Cyp4f16	1417277_at	1.9	4.40E-05	-1.5	8.88E-04
Cyp2d22	1419039_at	1.7	3.30E-04	3.5	1.12E-08
Cyp4b1	1416194_at	1.7	6.10E-04	3.3	3.68E-09
Cyp2d22	1419040_at	1.5	4.40E-03	3.1	2.71E-06
Cyp3a13	1419523_at	-1.2	5.20E-03	-1.4	3.52E-04
Cyp2j6	1417952_at	-1.4	1.20E-03	-1.7	1.42E-04
Cyp2s1	1428283_at	-1.4	4.60E-04	-1.2	7.50E-03
Akr1b8	1448894_at	13.4	1.60E-06	42.2	1.61E-10
Gstm4	1424835_at	2	1.50E-04	1.9	7.58E-03
Ephx1	1422438_at	1.7	1.90E-03	2.7	1.31E-05
Ephx2	1448499_a_at	1.5	3.30E-04	2.2	9.83E-08
Ugt1a2 /// Ugt1a6a /// Ugt1a10 /// Ugt1a7c /// Ugt1a5 /// Ugt1a9 /// Ugt1a6b /// Ugt1a1	1426261_s_at	1.5	6.50E-04	1.6	5.12E-06
Ugt1a2 /// Ugt1a6a /// Ugt1a10 /// Ugt1a7c /// Ugt1a5 /// Ugt1a9 /// Ugt1a6b /// Ugt1a1	1424783_a_at	1.5	9.70E-05	1.8	1.89E-06
Mgst1	1415897_a_at	1.4	2.30E-04	2.0	1.21E-07
Gstk1	1452823_at	1.3	3.60E-03	1.5	7.65E-04
Ugt1a2	1426260_a_at	1.3	4.70E-03	1.3	8.11E-04
Gstt2	1417883_at	1.2	4.20E-03	1.9	1.68E-06

Ugt2b34	1427961_s_at	-1.3	6.10E-03	-1.2	1.57E-02
Gstm5	1416842_at	-1.3	2.70E-03	1.2	1.59E-02
Sult1b1	1418940_at	-1.3	1.40E-03	-1.3	4.38E-04
Ugt2a3	1450133_at	-1.4	6.40E-04	-1.7	7.34E-06
Ugt2b34	1427960_at	-1.5	3.50E-04	-1.4	2.48E-04
Akr1c12	1422000_at	-1.5	4.50E-05	1.1	1.93E-01
Akr1c12	1450455_s_at	-1.7	9.40E-06	1.1	4.70E-01
Akr1c13	1418672_at	-1.7	5.60E-05	1.1	4.96E-01
Akr1c19	1455454_at	-1.9	2.10E-04	1.5	4.40E-03
Abca1	1450392_at	12.1	3.10E-05	8.8	1.71E-07
Abca1	1421839_at	10.9	4.20E-07	6.3	4.61E-08
Abca1	1421840_at	5.1	2.50E-06	4.1	4.15E-07
Abcd3	1416679_at	2.8	1.60E-07	2.8	4.29E-09
Abcg2	1422906_at	1.9	4.20E-06	1.5	4.77E-04
Abcd1	1418838_at	1.2	7.20E-03	1.1	4.60E-01

Presented are the fold changes and significance levels of all PPARα-dependently regulated barrier genes in the small intestine after acute (6hr) treatment with WY14643, and the corresponding fold changes observed after long-term (5 days) treatment with WY14643. Data on the acute effects of WY14643 are from the current manuscript, whereas the data from the 5-day experiment were taken from Bünger et al.

Listed are the gene symbols, corresponding Affymetrix probeset identifiers, fold changes (FC) and comparison p-values as determined in wild-type mice after acute exposure, and FC and comparison p-values as determined in wild-type mice after 5-day exposure to WY14643. Of all genes that were PPARα-dependently regulated after 6 hr exposure, 74% was also regulated after 5 days treatment with WY14643.

Reference:

Bünger M, van den Bosch HM, van der Meijde J, Kersten S, Hooiveld GJEJ, Müller M. Genome-wide analysis of PPARalpha activation in murine small intestine. Physiol Genomics. 2007;30(2):192-204.

PubMed ID: 17426115
