# Supplementary material for: PPARalpha-mediated effects of dietary lipids on intestinal barrier gene expression
Source: BMC Genomics. 2008 May 19;9:231. doi: 10.1186/1471-2164-9-231 (PMC2408604; doi:10.1186/1471-2164-9-231)
Supplement: Additional file 6 — Overlap of PPARα-dependently regulated barrier genes after acute treatment with WY14643, EPA, and DHA. [file 1471-2164-9-231-S6.pdf]

Additional data, table 6: Overlap of PPARα-dependently regulated barrier genes after acute treatment with WY14643, eicosapentaenoic acid, and docosahexanoic acid

Overlap WY14643 and EPA

Gene symbol	Probe set ID	FC WY	FC EPA
Slc16a13	1453056_at	10.7	1.5
Slc22a5	1440536_at	8.3	2.3
Slc22a5	1421848_at	7.7	3.2
Slc25a20	1423109_s_at	6.8	2.6
Slc25a20	1423108_at	5.6	2.3
Slc22a5	1450395_at	4.7	2.0
Slc27a2	1416316_at	2.2	1.6
Slc25a4	1455069_x_at	2.1	1.2
Slc25a4	1424562_a_at	1.9	1.2
Slc27a4	1424441_at	1.7	1.5
Slc25a10	1416954_at	1.6	1.5
Slc6a4	1417150_at	-1.4	-1.2
Slc5a9	1426634_at	-1.5	-1.4
Slc30a2	1427339_at	-1.5	-1.4
Slc7a8	1417929_at	-1.6	-1.6
Slc16a10	1436368_at	-1.7	-1.4
Slc5a9	1439494_at	-1.7	-1.7
Slc13a1	1430804_at	-2.1	-2.0
Cyp4a10 /// BC013476	1424853_s_at	1447.2	120.2
Cyp2c65	1429994_s_at	2.6	2.3
Cyp4f16 /// LOC545195	1430172_a_at	1.9	1.5
Cyp4f16	1417277_at	1.9	1.5
Cyp2d22	1419039_at	1.7	1.5
Akr1b8	1448894_at	13.4	3.2
Gstm4	1424835_at	2.0	1.8
Ephx1	1422438_at	1.7	1.6
Ephx2	1448499_a_at	1.5	1.4
Ugt1a2 /// Ugt1a6a /// Ugt1a10 /// Ugt1a7c /// Ugt1a5 /// Ugt1a9 /// Ugt1a6b /// Ugt1a1	1426261_s_at	1.5	1.3
Ugt1a2 /// Ugt1a6a /// Ugt1a10 /// Ugt1a7c /// Ugt1a5 /// Ugt1a9 /// Ugt1a6b /// Ugt1a1	1424783_a_at	1.5	1.2
Mgst1	1415897_a_at	1.4	1.3
Gstk1	1452823_at	1.3	1.3
Abca1	1450392_at	12.1	3.2
Abca1	1421839_at	10.9	2.9
Abca1	1421840_at	5.1	2.1
Abcd3	1416679_at	2.8	1.7

Overlap WY14643 and DHA

Gene symbol	Probe set ID	FC WY	FC DHA
Slc22a5	1421848_at	7.7	4.2
Slc22a5	1440536_at	8.3	3.1
Slc22a5	1450395_at	4.7	2.7
Slc25a20	1423109_s_at	6.8	2.4
Slc25a20	1423108_at	5.6	2.4
Slc16a13	1453056_at	10.7	1.8
Slc27a4	1424441_at	1.7	1.4
Slc4a4	1434096_at	-1.3	-1.3
Slc6a4	1417150_at	-1.4	-1.3
Slc44a4	1416596_at	-1.3	-1.5
Slc30a2	1427339_at	-1.5	-1.6
Slc17a5	1429116_at	-1.4	-1.7
Slc16a10	1436368_at	-1.7	-1.8
Slc5a9	1426634_at	-1.5	-1.8
Slc7a8	1417929_at	-1.6	-2.0
Slc5a9	1439494_at	-1.7	-2.0
Slc13a2	1418857_at	-2.1	-2.2
Cyp4a10 /// BC013476	1424853_s_at	1447.2	160.3
Cyp2c65	1429994_s_at	2.6	2.5
Cyp4f16	1417277_at	1.9	1.4
Akr1b8	1448894_at	13.4	5.1
Ephx1	1422438_at	1.7	1.9
Gstm4	1424835_at	2.0	1.8
Gstk1	1452823_at	1.3	1.5
Ugt1a2 /// Ugt1a6a /// Ugt1a10 /// Ugt1a7c /// Ugt1a5 /// Ugt1a9 /// Ugt1a6b /// Ugt1a1	1424783_a_at	1.5	1.3
Ugt1a2 /// Ugt1a6a /// Ugt1a10 /// Ugt1a7c /// Ugt1a5 /// Ugt1a9 /// Ugt1a6b /// Ugt1a1	1426261_s_at	1.5	1.3
Mgst1	1415897_a_at	1.4	1.2
Abcd3	1416679_at	2.8	1.8
Abcg2	1422906_at	1.9	1.3

Overlap EPA and DHA

Gene symbol	Probe set ID	FC EPA	FC DHA
Slc22a5	1421848_at	3.2	4.2
Slc22a5	1440536_at	2.3	3.1
Slc22a5	1450395_at	2	2.7
Slc16a13	1453056_at	1.5	1.8
Slc20a2	1457302_at	1.4	1.4
Slc27a4	1424441_at	1.5	1.4
Slc6a4	1417150_at	-1.2	-1.3
Slc4a4	1426432_a_at	-1.5	-1.5
Slc30a2	1427339_at	-1.4	-1.6
Slc16a10	1436368_at	-1.4	-1.8
Slc36a1	1428793_at	-1.6	-1.8
Slc5a9	1426634_at	-1.4	-1.8
Slc7a8	1417929_at	-1.6	-2
Slc5a9	1439494_at	-1.7	-2
Slc34a2	1416854_at	-2.7	-5
Cyp4a10 /// BC013476	1424853_s_at	120.2	160.3
Cyp2c65	1429994_s_at	2.3	2.5
Cyp4f16	1417277_at	1.5	1.4
Akr1b8	1448894_at	3.2	5.1
Hs3st1	1423450_a_at	2.1	2.7
Gstm3	1427473_at	2.4	2.6
Gstm3	1427474_s_at	2	2.1
Ephx1	1422438_at	1.6	1.9
Gstm4	1424835_at	1.8	1.8
Gstk1	1452823_at	1.3	1.5
Gsta1 /// Gsta2	1421041_s_at	1.2	1.3
Ugt1a2 /// Ugt1a6a /// Ugt1a10 /// Ugt1a7c /// Ugt1a5 /// Ugt1a9 /// Ugt1a6b /// Ugt1a1	1424783_a_at	1.2	1.3
Ugt1a2 /// Ugt1a6a /// Ugt1a10 /// Ugt1a7c /// Ugt1a5 /// Ugt1a9 /// Ugt1a6b /// Ugt1a1	1426261_s_at	1.3	1.3
Mgst1	1415897_a_at	1.3	1.2
Abcd3	1416679_at	1.7	1.8

Overlap WY, EPA and DHA

Gene symbol	Probe set ID	FC WY	FC EPA	FC DHA
Slc16a13	1453056_at	10.7	1.5	1.8
Slc22a5	1440536_at	8.3	2.3	3.1
Slc22a5	1421848_at	7.7	3.2	4.2
Slc25a20	1423109_s_at	6.8	2.6	2.4
Slc25a20	1423108_at	5.6	2.3	2.4
Slc22a5	1450395_at	4.7	2.0	2.7
Slc27a4	1424441_at	1.7	1.5	1.4
Slc6a4	1417150_at	-1.4	-1.2	-1.3
Slc5a9	1426634_at	-1.5	-1.4	-1.8
Slc30a2	1427339_at	-1.5	-1.4	-1.6
Slc7a8	1417929_at	-1.6	-1.6	-2.0
Slc16a10	1436368_at	-1.7	-1.4	-1.8
Slc5a9	1439494_at	-1.7	-1.7	-2.0
Cyp4a10 /// BC013476	1424853_s_at	1447.2	120.2	160.3
Cyp2c65	1429994_s_at	2.6	2.3	2.5
Cyp4f16	1417277_at	1.9	1.5	1.4
Akr1b8	1448894_at	13.4	3.2	5.1
Gstm4	1424835_at	2.0	1.8	1.8
Ephx1	1422438_at	1.7	1.6	1.9
Ugt1a2 /// Ugt1a6a /// Ugt1a10 /// Ugt1a7c /// Ugt1a5 /// Ugt1a9 /// Ugt1a6b /// Ugt1a1	1426261_s_at	1.5	1.3	1.3
Ugt1a2 /// Ugt1a6a /// Ugt1a10 /// Ugt1a7c /// Ugt1a5 /// Ugt1a9 /// Ugt1a6b /// Ugt1a1	1424783_a_at	1.5	1.2	1.3
Mgst1	1415897_a_at	1.4	1.3	1.2
Gstk1	1452823_at	1.3	1.3	1.5
Abcd3	1416679_at	2.8	1.7	1.8

Presented are the overlap of PPARα-dependently regulated barrier genes after acute treatment with the agonists for the combinations WY14643 and EPA; WY14643 and DHA; EPA and DHA; and WY14643. EPA and DHA. . Listed are the gene symbols, corresponding Affymetrix probeset identifiers, and the fold changes (FC) upon treatment with each agonist(s).
