# Supplementary material for: PPARalpha-mediated effects of dietary lipids on intestinal barrier gene expression
Source: BMC Genomics. 2008 May 19;9:231. doi: 10.1186/1471-2164-9-231 (PMC2408604; doi:10.1186/1471-2164-9-231)
Supplement: Additional file 7 — Additional confirmatory qPCR data. [file 1471-2164-9-231-S7.pdf]

Additional Table 7: Confirmation of microarray results, part 2

	WY14643		DHA	
**Gene symbol**	**FC (MA)**	**FC (qPCR)**	**FC (MA)**	**FC (qPCR)**
Slc22a5	**8.3***	**7.4** (2.2)*	**4.2***	**2.4** (0.5)*
Slc16a13	**10.7***	**6.0** (2.9)*	**1.8***	**1.5** (0.2)*
Abca1	**12.1 ***	**5.5** (1.9)*	**Nc**	**1.3** (0.3)
Akr1b8	**13.4***	**6.0** (1.6)*	**5.1***	**3.1** (1.6)*
Cyp4a10	**1447***	**319** (120.5)*	**160***	**44.9** (13.6)*
Abcd3	**2.8***	**4.7** (1.4)*	**1.8***	**2.3** (0.2)*

Microarray results were confirmed with qRT-PCR. FC = Fold change, MA = microarray, qPCR = quantitative PCR, nc = not changed. For the qPCR analysis: mRNA levels were standardized to cyclophilin; expression in the PPARα-null mice was arbitrarily set to 1. Significance was determined by a Bayesian t-test (array data) or unpaired student's *t*-test (qPCR data), *= p-value <0.05. Data are means ± standard error (n=4-5).
